# Bisoprolol reverses down-regulation of potassium channel proteins in ventricular tissues of rabbits with heart failure^☆^

**DOI:** 10.1016/S1674-8301(11)60037-7

**Published:** 2011-07

**Authors:** Xi Li, Tingzhong Wang, Ke Han, Xiaozhen Zhuo, Qun Lu, Aiqun Ma

**Affiliations:** a Department of Cardiovascular Medicine, the First Affiliated Hospital of Medical School, Xi'an Jiaotong University, Xi'an, Shaanxi 710061, China;; b Key Laboratory of Molecular Cardiology of Shaanxi Province, Xi'an, Shaanxi 710061, China;; c Key Laboratory of Environment and Genes Related to Diseases, Xi'an Jiaotong University, Xi'an, Shaanxi 710061, China;; d Department of Cardiovascular Medicine, the Second Affiliated Hospital of Medical School, Ningxia Medical University, Yinchuan, Ningxia Hui Autonomous Region 750001, China.

**Keywords:** heart failure, potassium channel, down-regulation, animal models

## Abstract

Remodeling of ion channels is an important mechanism of arrhythmia induced by heart failure (HF). We investigated the expression of potassium channel encoding genes in the ventricles of rabbit established by volume-overload operation followed with pressure-overload. The reversible effect of these changes with bisoprolol was also evaluated. The HF group exhibited left ventricular enlargement, systolic dysfunction, prolongation of corrected QT interval (QTc), and increased plasma brain natriuretic peptide levels in the HF rabbits. Several potassium channel subunit encoding genes were consistently down-regulated in the HF rabbits. After bisoprolol treatment, heart function was improved significantly and QTc was shortened. Additionally, the mRNA expression of potassium channel subunit genes could be partially reversed. The down-regulated expression of potassium channel subunits Kv4.3, Kv1.4, KvLQT1, minK and Kir 2.1 may contribute to the prolongation of action potential duration in the heart of rabbits induced by volume combined with pressure overload HF. Bisoprolol could partially reverse these down-regulations and improve heart function.

## INTRODUCTION

Heart failure is a clinical syndrome caused by various impairments in cardiac function. Sudden death, associated with arrhythmia, is responsible for approximately half of mortality rate among patients with heart failure[1]. Remodeling of ion channels and ion-transport function are an important mechanism of arrhythmia induced by diseased heart. The prolonged action potential duration (APD), associated with early after-depolarization (EAD), is a consistent finding in the ventricular myocytes of patients with heart failure or of animal models[2].

APD prolongation reflects an increased inward current or decreased outward current during the plateau of the action potential (AP)[3]. Potassium current as an outward current plays a pivotal role in APD. Transcriptional down-regulation underlies the molecular basis of changes in K^+^ currents. Down-regulation of Kv4.3 and Kv1.4 subunits underlies *I_to_* reduction in human failing hearts and rabbit failing hearts induced by ventricular tachycardia[4]–[6]. Reduced I_Ks_ appears to be due to transcriptional down-regulation of the α-subunit (KvLQT1) and β-subunit (minK) in rabbit heart failure model induced by ventricular tachycardia[5]. The change of *I_Kr_* units ERG has been consistent. Few studies have reported reduction of ERG[6]. Similar discrepancies exist in the study of I_K1_ unit Kir2.1[4],[7].

Furthermore, although the beneficial effects of β-blocker therapy have been known for quite a while[8]–[10], their exact mechanisms of action still remain incompletely understood. Studies report that most of ionic currents affected by β-adrenergic stimulation are also altered by β-blockers[11],[12]. In the present study, we explored the prolongation of the QT interval and down-regulation of K^+^ channel expression in rabbit heart failure model induced by volume-overload combined with pressure overload. The reversibility of these changes with bisoprolol was also demonstrated.

## MATERIALS AND METHODS

### Animal groups and materials

Thirty-one male New Zealand rabbits weighing 2.5 kg and 3.5 kg were obtained from Xi'an Jiaotong University Laboratorial Animal Center (Shaanxi, China). All animals received humane care in compliance with the Guide for the Care and Use of Laboratory Animals published by the US National Institute of Health (NIH Publication, No. 85-23, revised in 1996). Additionally, all experimental procedures were approved by the Care of Experimental Animals Committee of the First Affiliated Hospital of Medical School, Xi'an Jiaotong University, Shaanxi, China. After evaluation by echocardiography, the rabbits were randomly divided into three groups, namely, sham-operation rabbits as the control group (CTL, *n*=10), heart failure group (HF, *n*=10) and heart failure with bisoprolol treatment group (Biso, *n*=11).

All supplements were purchased as follows: Trizol from Sigma (USA), SYBR® Premix Ex Taq™ II from Takara (Dalian, China), rabbit BNP ELISA kit from Xitang Shanghai Biological Technology Co., Ltd. (Shanghai, China), bisoprolol fumarate tablets from Merck Serono (Germany), and 5F catheter from Cordis (USA).

### Establishment of heart failure model

Induction of heart failure model was performed according to the method of Den[13] with some modifications. In brief, following induction of anesthesia (sodium pentobarbital, 30 mg/kg, ip), a 5F catheter was introduced into the right carotid artery to induce aortic insufficiency by repeatedly moving the catheter to and fro across the aortic valve until the pulse pressure increased by >50%. After 3 w, rabbits were anesthetized to induce pressure overload as described above. Through a mid-abdominal incision the aorta was freed from the adjacent tissues at a point just above the renal arteries. A stainless steel rod (external diameter of 2.3 mm) was placed next to the aorta. A ligature was tied around the aorta and rod after which the rod was removed. This resulted in a reduction of the aortic diameter of approximately 50%. In sham operated rabbits, the right carotid artery and abdominal aorta was only freed from the adjacent tissues. After induction, the Biso group rabbits began to receive bisoprolol treatment (1 mg/kg/d) by gastric lavage for about 8 w.

### Evaluation of heart function

After 8 w of induction of pressure overload, rabbit heart function of the three groups was monitored by echocardiographic measurements. Left arterial diameter (LAD), aortic diameter (AOD), left ventricular end-diastolic dimension (LVDD), left ventricular end-systolic dimension (LVDS), left ventricular posterior wall (LVPW), interventricular septal thickness (IVST), ejection fraction (EF), fractional shortening (FS), and early diastolic filling to atrial filling velocity ratio of mitral flow (E/A) were measured. QT interval was measured using a standard lead II electrocardiogram (ECG). The QT interval was corrected (QTc) according to Carlsson's formula[14] for rabbits with QTc= QT-0.175(RR-300),and brain natriuretic peptide (BNP) in blood was measured using enzyme-linked immunosorbent assay (ELISA) kit.

### cDNA synthesis and quantitative real-time PCR analysis

Ventricular tissue RNA isolation and reverse transcription were carried out using the Superscript first-strand synthesis system (Takara, Dalian, China) according to the manufacturer's instructions. Specific primers for rabbit *Kir2.1*, *Kv4.3, Kv1.4, KvLQT1, minK, ERG* and *GAPDH* are shown in ***Table 1***. Real-time PCR was performed on an iQ5 Real-Time PCR Detection System (BIO-RAD, USA) using the SYBR® Premix Ex Taq™ II (Takara, Dalian, China) with an initial cycle of 95°C for 10 s followed by 50 cycles of 95°C for 5 s and 60°C for 31 s. Relative quantitation of target gene expression normalized to *GAPDH* was calculated according to the 2^−ΔΔC_T_^ method.

**Table 1:** Specific primers for rabbit target genes

Gene	Gene ID	Primer (5′-3′)	Size (bp)	TM (°C)
Kir2.1	NM001082198	F: ACTCCCCTGTGTAGTGCCAGA R: GCCTGGTTGTGAAGGTCAATG	179	62.0
Kv4.3	NC013681	F: GCCGCAGTAAGAAGACCACAC R: TTGGTCTCAGTCCGTCGTCTG	165	62.0
Kv1.4	NC013669	F: CAGCAGCAACAGGCCATGTC R: CTCCGCGAAATACACAGCCT	204	63.6
KvLQT1	NW003159627	F: GCCGCAGCAGTATGTCG R: CCTTCTCAGCAGGTACACGA	317	58.6
minK	NW003159355	F: GAGACGGCCCACCTACGG R: CGAAGAAGCCGAGCACCAT	112	57.0
ERG	NW003159353	F: TCGCACCATTAGCAAGATTC R: GGATGAGCCAGTCCCACA	263	62.0
GAPDH	NC013676	F: CTCTGGGGCTGTGGCGT R: GCTCGGGGATGACCTTGC	99	63.2

**Table 2:** Characteristics of heart function measurement in the three groups

	CTL	HF	Biso
LAD (mm)	7.73±0.41	9.16±1.77*	8.51±0.41*^#^
AOD (mm)	7.25±0.34	8.52±0.59*	8.20±0.64*^#^
LVDD (mm)	11.68±1.09	19.67±3.98*	15.10±1.21*^#^
LVDS (mm)	7.39±1.89	13.33±3.64*	7.70±1.32*^#^
LVPW (mm)	2.41±0.72	3.23±0.37*	2.70±0.27*^#^
IVST (mm)	2.16±0.81	3.42±0.54*	2.90±0.23*^#^
EF (%)	71.10±1.73	60.20±0.39*	67.20±0.25*^#^
FS (mm)	37.00±2.33	32.83±4.53*	34.00±4.57*^#^
E/A	2.65±0.35	2.00±0.53*	2.35±0.81*^#^
BNP (nmol/L)	2.83±0.35	4.65±0.47*	3.78±0.67*^#^

*P < 0.05 *vs* control group (CTL); ^#^*P* < 0.05 *vs* heart failure group (HF). AOD: aortic diameter; BNP: brain natriuretic peptide; E/A: early diastolic filling to atrial filling velocity ratio of mitral flow; EF: ejection fraction; FS: fractional shortening; IVST: interventricular septal thickness; LAD: left arterial diameter; LVDD: left ventricular end-diastolic dimension; LVDS: left ventricular end-systolic dimension; LVPW: left ventricular posterior wall.

**Table 3:** *In vivo* measurements

	Body weight (kg)	Heart rate	QT interval (ms)	QTc (ms)
CTL	3.38±0.26	284±11	110±9	125±7
HF	3.29±0.36	335±13**	114±6	137±5*
Biso	3.32±0.37	310±16*^#^	117±5	130±4*^#^

*P < 0.05 *vs* control (CTL), ^#^*P* < 0.05 *vs* heart failure (HF). QTc: corrected QT interval.

### Statistical analysis

Data were expressed as mean±SEM. Statistical comparisons between two groups were performed with one-way ANOVA test. Differences were considered significant at *P* < 0.05.

## RESULTS

### Characteristics of heart function of the three groups

Heart failure was confirmed by both echocardiographic examination and plasma BNP level test at 8 weeks after the pressure overload induction. As shown in ***Table 2***, compared with those of control rabbits, LAD, AOD, LVDD, LVDS, LVPW, IVST, and FS increased and EF decreased significantly in heart failure rabbits. Furthermore, plasma BNP level, a valuable marker of heart failure[15], also increased significantly in heart failure rabbits. After bisoprolol treatment, EF, FS and E/A of heart failure rabbits were increased significantly, compared with heart failure rabbits. In addition, BNP level decreased.

### ECG changes

***Table 3*** shows QT and QTc in electrocardiograhic results from the three groups. In the HF group, QT and QTc increased to (114±6) ms and (137±5) ms, respectively. However, QT and QTc increased to (117±5) ms and (130±4) ms, respectively, in Biso group.

### Expression of *I_to_* subunit mRNA

**Fig. 1:**
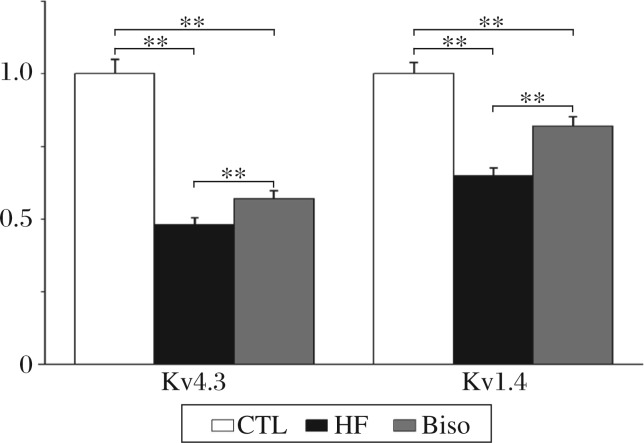
Histogram comparing relative amounts of the potassium channel isoform *Kv4.3* and *Kv1.4* mRNA in the ventricular myocardium of rabbits from the three groups. **P < 0.01. CTL: the control group; HF: the heart failure group; Biso: the bisoprolol group.

The expression of *I_to_* subunit mRNA was evaluated. As shown in ***Fig. 1***, the expression of *Kv4.3* was significantly down-regulated by 52% in the HF group (*P* < 0.01). In the Biso group, expression of *Kv4.3* was reduced by 44% (*P* < 0.01). Similar results were obtained for *Kv1.4* mRNA expression, reduction was 34% in the HF group (*P* < 0.01), but 17% in the Biso group (*P* < 0.01).

### Expression of *I_ks_* and *I_kr_* subunit mRNA

**Fig. 2:**
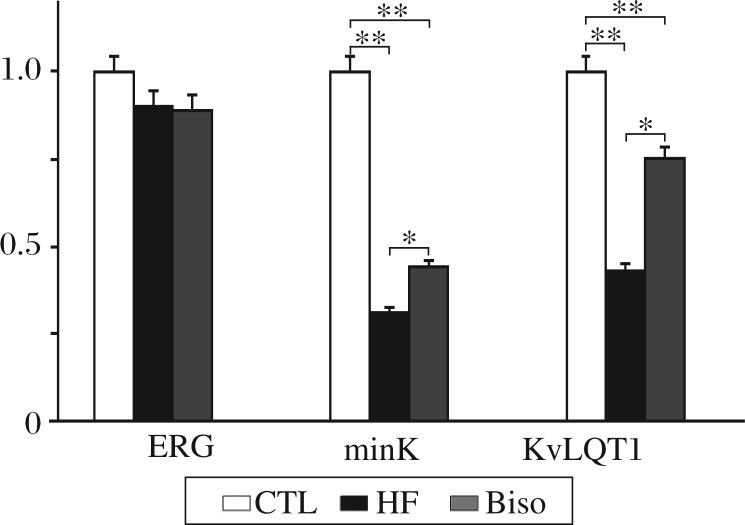
Real-time PCR results of KvLQT1, minK and ERG. Expression of KvLQT1 was 0.28±0.11 in the HF rabbits, 0.40±0.10 in the Biso group. Expression of minK was 0.39±0.09 in the HF rabbits, 0.68±0.08 in the Biso group. *P < 0.05, **P < 0.01. CTL: the control group; HF: the heart failure group; Biso: the bisoprolol group.

***Fig. 2*** shows *ERG*, minK and *KvLQT1* expression in the left ventricles of rabbits from the three groups. *KvLQT1* expression was reduced by 69% in the HF group and in the Biso by 56%. In addition, *minK* expression was reduced by 57% in the heart failure rabbits but 25% in bisoprolol rabbits. However, there were no significant differences in *ERG* expression among rabbits from the three groups.

### Expression of I_k1_ subunit mRNA

**Fig. 3:**
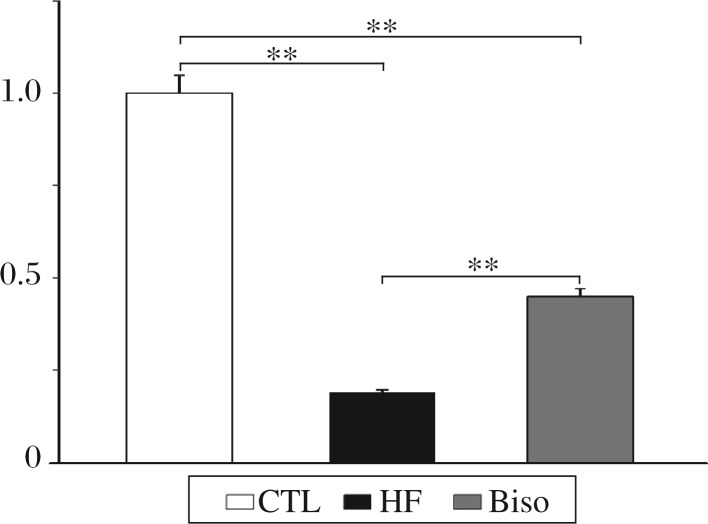
Real-time PCR results of Kir2.1. Relative fold of expression on Kir2.1 was 0.24±0.08 and 0.57±0.09 in the HF group and Biso group, respectively. **P < 0.01. CTL: the control group; HF: the heart failure group; Biso: the bisoprolol group.

*Kir2.1* expression in the left ventricles of rabbits from the three groups is shown in ***Fig. 3***. *Kir2.1* mRNA levels exhibited a trend toward reduced expression in failing ventricles. However, bisoprolol could reverse the change significantly. Compared with that of the control rabbits, the expression of *Kir2.1* was down-regulated by 81% in HF rabbits and by 55% in Biso group.

## DISCUSSION

In this study, we found that the expression of several genes encoding potassium channel isoforms (I_to_, I_Ks_ and I_K1_ subunits) was down-regulated in the heart of rabbits with volume combined with pressure overload heart failure. This may account for the prolongation of APD of ventricular myocytes. Treatment with bisoprolol, a β-blockers, could partially reverse these down-regulations and improve heart function.

Rabbits have a distinct plateau stage in action potential and a similar expression of K^+^ channels to human's. In our study, the heart failure model was induced in rabbits by volume-overload combined with pressure-overload procedure and confirmed by echocardiographic examination and plasma BNP level test. The model with both systolic and diastolic dysfunctions has a similar pathophysiology to a human one, including ventricular hypertrophy and dilatation, combined with decreased EF and FS 8 weeks after surgery by echocardiography. Plasma BNP was increased significantly and QTc was prolonged in ECG of failing heart.

Potassium currents play a key role in the formation of APD[2]. Transient outward current (I_to_, encoded by Kv4.3 and Kv1.4) is the main current in phase 1 early rapid repolarization. Phase 3 is a late repolarization phase of APD dominated by I_Kr_ (encoded by ERG and minK) and I_ks_ (encoded by KvLQT1 and minK). I_K1_ (encoded by Kir2.1) is a determinant factor for maintenance of resting potential in phase 4. APD is shown as QT in ECG clinically. Prolonged QTc is consistent with findings in studies of human and animal heart failure models[16],[17].

Studies have shown that repolarization abnormalities and QT/APD prolongation may be due to changes of K^+^ currents induced by heart failure[6],[18]. In our study, there was a reduction in mRNA level of Kv4.3 by 52%, Kv1.4 by 34 %, KvLQT1 by 69%, minK by 57% and Kir2.1 by 81% in the left ventricles from the heart failure rabbit. However, ERG mRNA was not observed with great changes. Such remodeling of K^+^ channel expression was accompanied with QTc prolongation. Our data are in agreement with those of previous studies in the failing ventricles from human and animal models. In rabbit failing heart induced by tachypacing, studies showed that *Kv4.3, Kv1.4, KvLQT1* and *minK* mRNA were reduced[6]. In canine heart failure model induced by tachypacing, a significant decrease in *Kv4.3* mRNA[19] has been found. In the human failing heart, *Kir2.1* and *Kv4.3* mRNA was measured with a striking decrease by gene microarray analysis, while *KvLQT1, minK* and *ERG* mRNA were found[4].

Prolongation of QT or APD, reflecting impaired repolarization, is a consistent finding in previous studies[20],[21]. Early after-depolarizations (EADs) and delayed after-depolarizations (DAD) are the heart failing-induced arrhythmias which are associated with remodeling of K^+^ currents. EAD commonly occurs in phase 2 and phase 3 of APD. EAD is the result of reduction of I_Ks_, I_Kr_, I_to_ and I_K1_[2],[21]. DADs are favored by increased Na^+^-Ca^2+^ exchange and reduced IK1. Remodeling of K^+^ currents plays a key role in cardiac arrhythmias associated with heart failing. The decrease of mRNA expression of K^+^ channels subunits underlies the molecular down-regulation of K^+^ currents[2].

Bisoprolol has been confirmed to be beneficial for shortening prolonged QT induced by heart failure in clinical trials and animal experiments[23],[24]. Our present study is in agreement with previous reports. Our previous study on heart failure rats showed a significant reduction of I_to_ and I_K1_ and expression of I_to_ and I_K1_ at the protein level. We also found that bisoprolol could reverse the down-regulation of I_Kr_, I_to_ and I_K1_. The interesting findings showed in our study are that bisoprolol could reverse down-regulation of K^+^ current expression. These findings may underlie the molecular mechanism for antiarrhythmia of bisoprolol.

Ion-channel function is regulated by channel phosphorylation state, neurotransmitters, and interactions with other channels and transporters. In classic β1-adrenoceptor-mediated signaling in cardiomyocyte, protein kinase A (PKA) passway activated via secondary messenger cAMP can phosphorylate many proteins involved in ion channels resulting in changes of their kinetic properties.β-Adrenergic stimulation via cAMP-dependent PKA mechanism enhances I_K1_ and I_Ks_ current[25]. while decreases HERG(α-subunit of I_Kr_) current by 19%-40%[26]. However, exact mechanism of β-blockers modulating the remodeling of potassium channels in cardiomyocyte were still unclear. Experiments with isoproterenol and β-blockers demonstrated that IK is affected by PKA phosphorylation[27]. The results also suggested β-blockers suppress adrenergic stimulation and alter PKA to regulate potassium channels.

In summary, our data demonstrated that down-regulated expression of potassium channel isoforms Kv4.3 and Kv1.4 (encoding I_to_ subunit), KvLQT1 and minK (for I_K_) and Kir 2.1 (for I_K1_) may contribute to the prolongation of APD in the heart of rabbits with volume combined with pressure overload heart failure. Additionally, treatment with bisoprolol, a β-blockers, can partially reverse these down-regulations and improve heart function.
